# COVID-19 Vaccines: Current Conditions and Future Prospects

**DOI:** 10.3390/biology10100960

**Published:** 2021-09-26

**Authors:** Tarek Zieneldien, Janice Kim, Jessica Cao, Chuanhai Cao

**Affiliations:** 1 Department of Pharmaceutical Science, Taneja College of Pharmacy, University of South Florida, Tampa, FL 33612, USA; tarekz@usf.edu (T.Z.); janicekim1@usf.edu (J.K.); 2 Department of Natural Sciences, Wiess School of Natural Sciences, Rice University, Houston, TX 77005, USA; jessica.a.cao@rice.edu

**Keywords:** COVID-19, lipid nanoparticles, mRNA vaccine, adenovirus, angiotensin-converting enzyme 2 (ACE2), spike protein

## Abstract

**Simple Summary:**

The coronavirus disease 2019 (COVID-19) pandemic, caused by the novel severe acute respiratory syndrome coronavirus 2 (SARS-CoV-2), was first encountered in December of 2019 in Wuhan, China. As of now, there have been over 200 million infections and 4 million deaths attributed to the virus. Due to this, it has been a priority to find an effective preventative measure, and numerous vaccines have been developed. Although the developed vaccines share the target of blocking viral entry by the spike protein, their pharmacology and efficacy differs. As such, the mechanism of action and the elicited immune response of the most common COVID-19 vaccines have been compared to help determine which vaccine is most efficacious and is best suited to prevent reinfection and address viral mutations.

**Abstract:**

It has been over a year since SARS-CoV-2 was first reported in December of 2019 in Wuhan, China. To curb the spread of the virus, many therapies and cures have been tested and developed, most notably mRNA and DNA vaccines. Federal health agencies (CDC, FDA) have approved emergency usage of these S gene-based vaccines with the intention of minimizing any further loss of lives and infections. It is crucial to assess which vaccines are the most efficacious by examining their effects on the immune system, and by providing considerations for new technological vaccine strategies in the future. This paper provides an overview of the current SARS-CoV-2 vaccines with their mechanisms of action, current technologies utilized in manufacturing of the vaccines, and limitations in this new field with emerging data. Although the most popular COVID-19 vaccines have been proven effective, time will be the main factor in dictating which vaccine will be able to best address mutations and future infection.

## 1. Introduction

The first outbreak of the COVID-19 infection occurred in Wuhan, China in 2019 where many patients had symptoms that were similar to respiratory infections and this infection rapidly spread [1]. The COVID-19 pandemic has catastrophically swept across the world, resulting in over 200 million infections and 4.5 million deaths (as of 30 August 2021) [2]. The unpredictable nature of the pandemic and lack of preparedness against infectious diseases on a global scale has led to business shutdowns, widespread economic recession, and millions of jobs lost [3]. The rapid spread of the virus has largely been attributed to a rise in globalization and international collaboration, coupled with the significant achievements in controlling infectious diseases in the past, which has encouraged society to ignore the dangers of emerging infectious diseases [4,5]. Furthermore, the pandemic has drastically changed everyday life, with masks, physical distancing, and remote learning and working becoming the norm for millions of people [6,7]. Nonetheless, numerous countries have implemented resilient measures, such as emergency provisions, to curb the spread of the pandemic [8]. Even more, there has been coordinated planning between nations to distribute access to vaccines, thereby allowing impoverished nations and their citizens to receive the vaccine [9].

Currently, there have been emergence of variants for SARS-CoV-2 worldwide. The current variants that are of concern which have characteristics of increased transmissibility and increased virulence include the Alpha, Beta, Gamma, and Delta variants [10]. The variants of interest which have an emerging risk against global health but are still being monitored include the Eta, Iota, Lambda, and Kappa variants [10].

Coronaviruses are known to infect many animals, have the largest RNA genome and contain four subfamilies which include alpha, beta, gamma, and delta coronaviruses [11]. All four subfamilies have the commonality of being from a zoonotic origin where the alpha and beta coronaviruses emerge from bats and the gamma and delta coronaviruses emerge from birds [11]. The most recent occurrences of coronavirus infections occurred in 2001 and 2012 with both being from zoonotic origins. The 2001 epidemic occurred with severe acute respiratory syndrome coronavirus (SARS-CoV) and in 2012 was the Middle East respiratory syndrome coronavirus (MERS-CoV) and while both epidemics resulted in severe respiratory infections, the novel SARS-CoV-2 infections have surpassed both SARS-CoV and MERS-CoV [12]. All three of the coronavirus epidemics mentioned belong to the beta-coronaviruses which contain envelope proteins that are utilized for viral infection [11]. The important envelope proteins include the spike (S) protein, envelope (E) protein, and the membrane (M) protein, which mediates viral entry into the host cell [13].

### 1.1. The COVID-19 Virus: Infection and Symptoms

SARS-CoV-2 is a single-strain, positive RNA virus with a relatively short replication time that is highly pathogenic and infectious. SARS-CoV-2 appears to preferably infect respiratory tract cells, but has also been detected in nearly all of the human organs, such as the liver, pharynx, lungs, heart, digestive system organs, and kidneys. To illustrate, patients with COVID-19 have reported signs of kidney injury, which could be explained by renal tropism [14]. Due to this, it could be suggested that organotropism could influence the course of COVID-19 progression [14].

**Figure 1:**
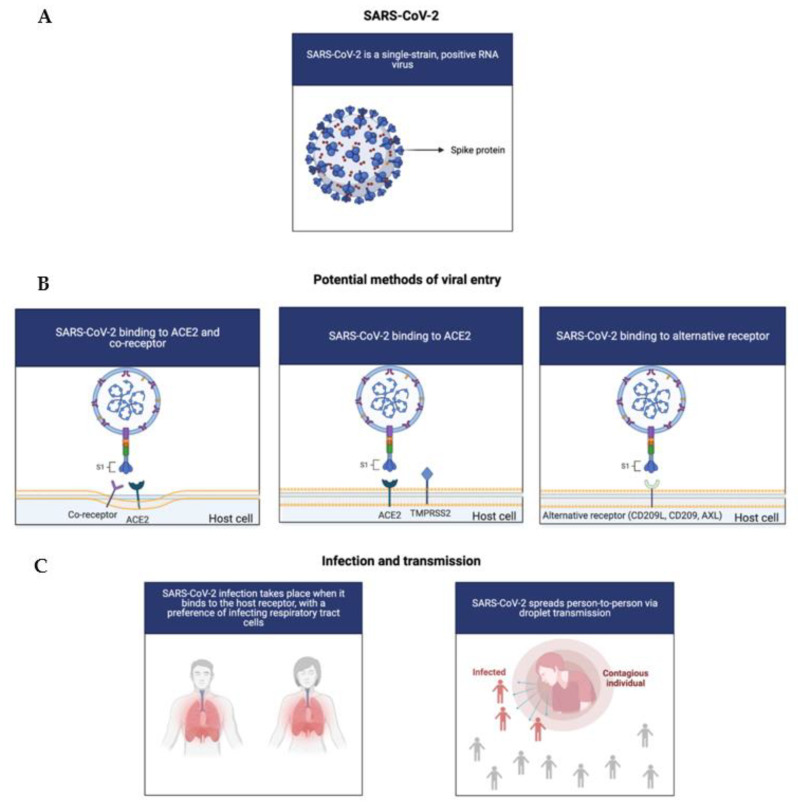
**COVID-19 Infection and Transmission.** (**A**): Illustration of SARS-CoV-2. (**B**): The potential mechanisms of infection for SARS-CoV-2, which preferentially infects respiratory tract cells. (**C**): COVID-19 transmission when an infected individual is in close contact with others, spreading via droplets from talking, breathing, coughing, or sneezing.

Infection occurs when the virus binds to the host receptors via the receptor binding domain (RBD) of the spike protein (S), which thereby mediates membrane fusion and viral entry [12]. Then, transmembrane protease serine 2 (TMPRSS2) and FURIN serve to cleave the S protein into the N-terminal S1 subunit and the C-terminal S2 subunit [15]. Although angiotensin-converting enzyme 2 (ACE2) functions as the entry point for the virus, the potential of co-receptors and novel receptors has also been considered, due to the fact that ACE2 expression is seemingly low in a plethora of human tissues, such as the respiratory tract [16]. Recently, various studies have elucidated the potential of tyrosine-protein kinase receptor UFO (AXL), CD147, CD209L, and CD209 as alternative receptors and co-receptors (Figure 1) [15,17,18]. To elucidate, overexpression of AXL has been shown to facilitate viral entry of both authentic SARS-CoV-2 and pseudoviral SARS-CoV-2 in HEK293T cells, accentuating the complexity of SARS-CoV-2 viral entry [15]. Nonetheless, given these mechanisms of infection, targeting the S protein or the RBD appears to be an advantageous approach for treatment development.

In general, the virus spreads via the droplet transmission of symptomatic individuals, with airborne droplets arising from breathing and speaking, providing significant risk since they are in respirable size ranges [1,19]. An asymptotic individual may also spread infection as the incubation period of the virus is between 2 and 14 days [1]. The virus also primarily manifests as a lower respiratory tract disease, which leads to respiratory distress. Individuals with preexisting health conditions—obesity, cardiomyopathies, and diabetes—have reported a higher risk of experiencing severe COVID-19 symptoms due to potentially impaired immune systems [20,21,22]. The most common clinical manifestations for this disease include shortness of breath, fever, cough, and fatigue while the less common symptoms include headache, vomiting, and dizziness [23]. These symptoms are similar to many respiratory infections. As the infection progresses, deadly complications include respiratory failure and pneumonia, which can ultimately lead to death [1].

### 1.2. Treatment and Vaccine Approaches for COVID-19

Various treatments for COVID-19 have been explored, such as antivirals like remdesivir, corticosteroids such as dexamethasone, and other drugs such as tocilizumab, ivermectin, and chloroquine. However, these are novel treatments and their effectiveness against COVID-19 has not been validated through clinical trials [24,25]. Ivermectin only showed antiviral properties after large doses, which is unsafe to humans as it is over the conventional usage limit and may induce adverse side effects such as seizures, overdose, and psychosis [26]. Moreover, chloroquine showed severe adverse effects and drug interactions, making it unsuitable as an effective COVID-19 treatment [26]. Unfortunately, since COVID-19 is a novel virus, scientists remain uncertain as to how long infected individuals should use such pharmaceuticals. Moreover, clinical results have indicated that potentially effective treatments for this infectious disease are anti-sera treatments, with vaccines being an ideal preventative measure [27]. Anti-sera from recovered patients have been used to treat those with severe respiratory conditions, but due to its limited supply and scarce evidence on effectiveness, the most promising and effective solution is still dependent on a preventative vaccine [28,29,30,31,32]. Furthermore, implementing mass vaccination is crucial, as high compliance rates are essential to developing herd immunity. This is evident in the prevalence of other diseases such as measles, smallpox, and poliomyelitis, which have been nearly or completely eradicated via the widespread usage of vaccines [33]. Currently, there are numerous types of vaccines that have been developed for COVID-19, including DNA vaccines, mRNA vaccines, and recombinant protein (spike) vaccines [34,35,36,37,38]. In DNA vaccines, the genetic material needs to pass through the nucleus to create messenger RNA, which then allows the formation of protein in the cytoplasm [39]. However, mRNA vaccines are advantageous in that they are able to bypass this step, making the process more potent, and allows for rapid development of the functional proteins in the cytoplasm [40,41]. Since each type of vaccine has its advantages and disadvantages, it is pivotal to provide a clear explanation of each vaccine type so the public will be more informed on which vaccine to receive.

### 1.3. The Role of Antibody and T Cell Responses in Fighting COVID-19

There are two mechanisms of immune response in fighting viral infection: antibody against infectious agent and antigen specific T cell response [42]. Most vaccines focus on the antibody response, and T cell responses are largely neglected. Although it is currently difficult to determine the exact antibody levels and duration after infection, evidence suggests that individuals who have recovered from COVID-19 have developed a favorable immune response regarding their memory CD4+ and CD8+ T cells [20]. In some patients, memory CD4+ and CD8+ T cells have generated responses for numerous COVID-19 proteins, such as the nucleoprotein and spike proteins [20]. Although the antibodies to the nucleoprotein are improbable to neutralize COVID-19, there have been cases in which they have produced satisfactory protection, such as with the mouse hepatitis virus (MHV), which is also a coronavirus [43,44]. The most prominent of the antibodies was IgG2a, showing that protection could be conceived via Fc-mediated effector functions instead of a virus neutralization that is solely direct [43,44,45,46,47,48].

During COVID-19 infection, IgG and IgM antibodies are often detected in the first two weeks after the onset of symptoms. Furthermore, antibodies that are able to bind to the S1 domain RBD can effectively block its binding to ACE2. On the other hand, antibodies that bind to different regions of the S1 and S2 domains can inhibit the change of the S protein conformation and thereby prevent fusion to the membrane [44,45,49,50,51].

In older individuals, COVID-19 has manifested with greater severity, as is evident by the higher mortality rates in older populations [52,53]; this corresponds with the general consensus that older individuals have less robust immune systems than their younger counterparts. Older COVID-19 patients have also exhibited graver cases of lymphopenia, which affects CD4+ T cells, CD8+ T cells, natural killer cells (NKCs), and B cells [54,55]. Furthermore, cases of spleen and lymph node necrosis have been reported in older COVID-19 patients, which is particularly physiologically detrimental due to their already weakened immune systems [56].

When examining the immune response generated by CD8+ T cells, it is inconclusive whether T cell hypoactivation or hyperactivation occurs [20]. In general, CD8+ hyperactivation manifests with elevated cytotoxicity and NKC-related markers, while hypoactivation displays as the opposite [20]. Data from recovered COVID-19 patients show that memory CD8+ T cells were found in approximately 70% of the recovered individuals, with 100% of the recovered individuals having memory CD4+ T cells [20,44]. However, this is preliminary data is therefore inconclusive in determining whether these memory T cells would yield preventive immunity. Therefore, it is necessary to identify the T cell response patterns to evaluate beneficial individualized therapies.

### 1.4. T-Cells and Pre-Existing Cross-Reactive Immunity

When examining the COVID-19-reactive T-cells in patients and unexposed persons, it has been seen that CD4+ T cells that can recognize the COVID-19 S protein are present in 35% of the unexposed individuals, and at least 40% of the unexposed individuals have CD4+ T cells that are cross-reactive to other COVID-19 proteins than just the S protein [57,58]. In general, this illustrates that there is cross-reactivity among CD4+ T cells for prevalent coronaviruses that affect humans and COVID-19, with CD4+ T cells from COVID-19 patients impartially recognizing both the S1 and S2 subunits of SARS-CoV-2, while the cross-reactive CD4+ T cells recognize the S2 subunit [58]. Thus, common coronaviruses such as HKU1, NL63, OC43, and 229E that infect humans could cause a certain extent of prior cross-reactive immunity to the COVID-19 antigens, granting different immune responses and severity of disease in the general population, although their specific biological role remains undetermined [44,59]. In fact, serum IgG antibodies have been estimated to be found in greater than 90% of the population for these common coronaviruses [59]. This is crucial to acknowledge during vaccination as it allows the effects of vaccination-boosted cross-reactive responses to be considered when examining the defensive immunity that was induced by the actual vaccine [44].

Consequently, unexposed persons have been found to contain CD4+ T cells that recognize the COVID-19 S2 protein, while the infected individuals seem to have CD4+ T cells that do not discriminate between the S1 and S2 protein subunits [44,58]. Furthermore, the S2 protein of the 229E/OC43 coronaviruses has been found to be cross-reactive with CD4+ T cells of COVID-19 infected individuals [44,58]. Due to this, the prior cross-reactive immunity could hinder the potency of live attenuated vaccines.

### 1.5. Current and Most Common COVID-19 Vaccines

Vaccines work through three major steps: antigen phagocytosis and presentation; T cell activation and cytokine production; cytokine stimulation of B cells to differentiate into plasma cells [60]. Therefore, both B and T cells are involved in a vaccine related immune response. As such, antigen selection and therapeutic target are very critical for the success of a vaccine. The most popular and successful target consists of blocking the viral entry by the antibody, so S protein, particularly the RBD, will be the therapeutic target for COVID-19 since it prevents binding to the host receptors. The S protein, which has two domains (S1 and S2), also serves as the most significant target of the neutralizing antibodies [61]. The S1 domain includes the RBD, which ultimately binds to the ACE2 receptor with a relatively high affinity [62].

The first vaccine that was authorized for emergency use against COVID-19 was Pfizer’s mRNA vaccine, which has a prospective efficacy of more than 90%, according to the interim analysis [63]. This vaccine is administered via intramuscular (IM) injection and consists of a lipid-enclosed, nucleoside-modified mRNA that encodes the configuration of a mutated COVID-19 spike protein [64]. The Pfizer vaccine consists of two 30 μg doses, with the second dose administered three weeks after the initial vaccination [65]. The lipid nanoparticles allow mRNA of the S gene of COVID-19 to deliver into the host cell, resulting in expression of the COVID-19 spike protein antigen. This then elicits an immune response to the spike protein, which provides the body with protection against the virus [66]. Moderna has similarly developed an mRNA vaccine with a nucleoside-modified messenger RNA and a mutated spike protein that allows for further stability, since the two mutations involve the original amino acids interchanged with proline [35]. The Moderna vaccine also depends on the lipid nanoparticles for delivery and consists of two 100 μg doses, with the second vaccine being administered four weeks after the initial IM vaccination [67]. The lipid nanoparticles allow for the nucleoside-modified mRNA delivery into the host cell, which in turn expresses the COVID-19 spike protein antigen. The antibodies that are consequently produced are specific to the virus, allowing for future protection [68]. Unfortunately, the distribution of both vaccines is difficult in developing countries, particularly those with hotter climates, as multiple dose vials must be stored between −80 °C to −60 °C (Pfizer) or between −25 °C and −15 °C (Moderna) [68,69].

Unlike Pfizer and Moderna, Oxford/AstraZeneca, Gamaleya, and Johnson & Johnson have created adenovirus vector vaccines. The Oxford/AstraZeneca vaccine utilizes a viral chimpanzee non-replicating adenovirus vector and has a reported efficacy of 81.3% when both doses are administered [70,71]. This vaccine has the complete coding sequence of the COVID-19 spike protein, as well as a sequence of a tissue plasminogen activator [72,73]. After IM delivery, the chimpanzee adenovirus enters and infects the cell to produce the COVID-19 virus in the cytoplasm of the cell and upon infection, it triggers the immune system to begin producing antibodies and immune B and T cells for protection against future infections that may occur [74,75]. In the trials for the AstraZeneca vaccine, only 10% of vaccine recipients reported noticeable side effects, with headache, nausea, and muscle pain being the most common [76]. In terms of adverse side effects, there have been approximately 194 reports of anaphylaxis out of more than 9 million vaccinations [77].

The Gamaleya vaccine is a viral vector vaccine that utilizes two different adenoviruses found in humans. The two viral vectors are recombinant adenoviruses and are administered at different times. The recombinant type 26 adenovirus is administered first, followed by the recombinant type 5 adenovirus, which is given as a booster 21 days later [74,78,79]. This vaccine employs a heterologous approach, which has the potential of inducing a broader immune response, and has a reported efficacy of 91.6% after both doses, according to the interim analysis [80,81]. Furthermore, in the Gamaleya trials, all of the participants generated antibodies against the COVID-19 spike protein. In the Gamaleya phase I–II safety trials, the vaccine did not cause any severe adverse effects, but caused headache, hyperthermia, asthenia, and muscle pain [81]. However, there have been some concerns regarding the Gamaleya vaccine as documentation revealed one of the two doses contained adenoviruses that were capable of replication, which could potentially be dangerous to the recipients [82].

Unlike the aforementioned vaccines which all require two doses, the latest viral vector vaccine to be released is the single-shot Johnson & Johnson (J&J) vaccine. This vaccine utilizes a non-replicating recombinant adenovirus, the type 26 recombinant adenovirus, which expresses the COVID-19 spike protein [83]. The J&J vaccine also has mutations that stabilize the spike protein by having the normally found amino acids interchanged with prolines [84,85]. Clinical trials for the J&J vaccine began in June of 2020, and the phase III trials had more than 43,000 ethnically diverse participants; this vaccine was ultimately determined to be 66% effective [86]. All these viral vector vaccines are unable to replicate, which is explained by the fact that certain genes that are essential for replication were deleted and exchanged by genes that code for the COVID-19 spike protein [83].

### 1.6. Additional COVID-19 Vaccines

The Sinopharm vaccines are a two-dose, inactivated virus vaccines that are administered via IM injection [87,88]. These vaccines contain an aluminum hydroxide adjuvant to modulate the immune system, and are relatively advantageous because they can be stored and distributed at standard refrigerated temperatures [89]. However, Sinopharm’s analysis of their vaccines shows an efficacy of around 79% for BBIBP-CorV and 72.8% for WIBP-CorV, which is much lower than that of Pfizer and Moderna’s vaccines [90,91]. Nonetheless, available data shows that when both doses are administered, an efficient humoral immune response is generated in all recipients [88]. The present data also showed that their vaccines can produce significant levels of neutralizing antibody titers in rhesus macaques, pigs, mice, guinea pigs, and rats [88]. While the data at present supports the vaccine efficacy, there have been scattered data regarding vaccines from China since this is a quickly moving field, hindering the transparency of these vaccines, which may lead to hesitation from individuals [92].

Recently, a protein subunit vaccine has been made by Novavax, with a reported vaccine efficacy of 89.3% [93]. This vaccine was created by engineering a baculovirus that contains a COVID-19 altered spike protein gene which encodes vaccine antigens, and allowing it to attack cultured Sf9 insect cell lines *(Spodoptera frugiperda*) [94]. Upon infection of the Sf9 insect cells, the antigens are expressed creating the recombinant nanoparticle containing S protein configurations [95]. The adjuvant for this vaccine is saponin-based [96]. Furthermore, the formulation can be easily distributed, as it is stable from 2 °C to 8 °C [97]. Another adjuvanted protein subunit vaccine, Zifivax, has been developed in China, but requires three doses which might lead to some individuals not completing their vaccine regimen [98].

In regard to peptide-based vaccines, the VECTOR center of Virology has developed EpiVacCorona, which contains three conjugated chemically synthesized peptides that are aided by a carrier a protein [99]. EpiVacCorona contains an aluminum hydroxide adjuvant and utilizes the IM injection route for drug delivery [99]. Although there have been some concerns in regard to the immunogenicity data, EpiVacCorona has been licensed for use in Russia, Turkmenistan, and Belarus [100].

### 1.7. Lipid Nanoparticles and the Lipid mRNA Vaccine of COVID-19

Solid lipid nanoparticles (SLNs or sLNPs) are a rapidly developing technology, with many applications in the pharmaceutical industry, including novel drug delivery [101]. Currently, its application in cancer treatments has become popular with monoclonal antibodies, therapeutic vaccines, and immunotherapies taking advantage of utilizing the lipid-based drug delivery system, including lipid-based nanoparticles, solid lipid nanoparticles, and nanostructured lipid carriers [102]. The lipid based nanoparticles provide transport of the materials without causing toxicity, increasing the control of drug release, and the systems of delivery are compatible with different pH sensitivities [103]. Moreover, these lipid nanoparticles can be utilized in adjuvants for protein-based vaccines [104] These nanoparticles have been perceived as potentially promising drug carriers due to their structural and compositional benefits when compared to conventional formulas [105]. The widespread applications of SLNs have rapidly developed as a result of their numerous advantages, including targeted drug release, affordability, and enhanced stability of pharmaceutical products. Research relating to SLNs has been on the rise since the start of the COVID-19 pandemic, and several mRNA vaccines for COVID-19 have been produced by utilizing lipid nanoparticles as their drug delivery system. However, an issue that has arisen with using SLNs to deliver nucleic acids is that both lipids and nucleic acids have negative charges, and are therefore not ionizable [106].

Extensive research has consequently been conducted to find a solution, which has led to the creation of ionizable cationic lipids that bind to mRNA [107]. The ionizable lipid maintains a neutral pH, eliminating any positive charges, and cholesterol limits the protein interactions that SLNs face [41]. Furthermore, SLNs shield the delicate mRNA from the enzymes, allowing the mRNA to reach the cells without being degraded by the body’s enzymes [108]. Due to the importance of these functions, choosing the appropriate lipid is crucial for the creation of an effective lipid nanoparticle for the delivery of the COVID-19 mRNA vaccine. This includes ionizable or cationic lipids, which would allow the SLNs to remain undetected throughout the body due to the lipids’ neutral surface charge [108].

This technology has been utilized by both Moderna and Pfizer-BioNTech (Pfizer) mRNA vaccines, which use ionizable cationic lipids that encapsulate the nucleoside-modified mRNA encoding a spike protein of SARS-CoV-2 [109]. The Moderna vaccine utilizes the SM-102 ionizable lipid, while Pfizer uses ALC-0315 [110]. During the early stages of vaccine development, Moderna was conducting testing on the ionizable lipid MC3 but replaced it with the SM-102 lipid due to the slow degradability and low potency of MC3 [111]. Moreover, the MC3 lipid was used in siRNA products, and it was reported that slow lipid degradability led to the potential of accumulation toxicity with repeated doses [112]. The new SM-102 lipid has an ethanolamine ionizable head, which allows for an increase in branching, resulting in a greater membrane-disrupting capacity when paired with the endosome [113]. The Pfizer vaccine utilizes the ALC-0315 lipid as the delivery system, which contains two nucleoside-modified mRNA, one unmodified mRNA, and one self-amplifying mRNA [114]. In utilizing lipid nanoparticles, hypersensitivity has been reported in some cases including contact dermatitis from the interactions between the LNPs and the immune systems and anaphylaxis due to the polyethylene glycol lipid (PEGylated lipid) in the mRNA vaccines [115,116,117].

### 1.8. The Unique Pharmacokinetic/Pharmacodynamic (PK/PD) Property of Lipid mRNA Particles

From a chemical perspective, both ionizable and cationic lipids can serve as a medium for SLN delivery. However, cationic lipids have been reported to be toxic in clinical therapeutics, with concerning accumulations in the liver and spleen that have led to inflammatory responses and immune cell activation [118,119]. To limit the toxicity of this drug delivery method, ionizable cationic lipids are preferred. Ionizable cationic lipid nanoparticles penetrate cells through receptor-mediated endocytosis [120]. During this process, lipid nanoparticles end up in the acidic endosomes and gain a positive charge, which triggers the lipid nanoparticle to release its RNA into the cell [121]. The mRNA vaccines rely on lipid nanoparticles to safely transport mRNA to its specific cellular compartment [122]. Since mRNA is rapidly degraded if left unprotected, SLNs enable the mRNA to reach the cytoplasm of the host, where the mRNA undergoes cellular translation to produce proteins [123]. This is advantageous for mRNA vaccines because mRNA is delivered to the correct, precise location, allowing for controlled release and thereby reducing the risk of toxicity [124].

The mRNA vaccines for COVID-19 contain mRNA that encodes the spike protein of SARS-CoV-2. These vaccines are administered via intramuscular (IM) injections, which provides access to immune cells, as there is a vast network of blood vessels available in muscles [125]. The IM injection also allows for the vaccine to induce a strong, prolonged expression of the protein in immune cells from the mRNA vaccine, particularly since there are antigens in muscle tissues and lymph vessels near the muscle where the antigens can be processed [126]. In eliciting an immune response to the virus, the mRNA vaccine targets T and B cells which generate antibodies for protection and eliminates infected cells [127,128]. The mechanism for the immune response after the uptake of the lipid nanoparticles begins with mRNA being translated into proteins, then the individual’s immune system will generate and optimize an immune response to the targeted proteins in SARS-CoV-2 through potent neutralizing antibodies [127].

**Figure 2:**
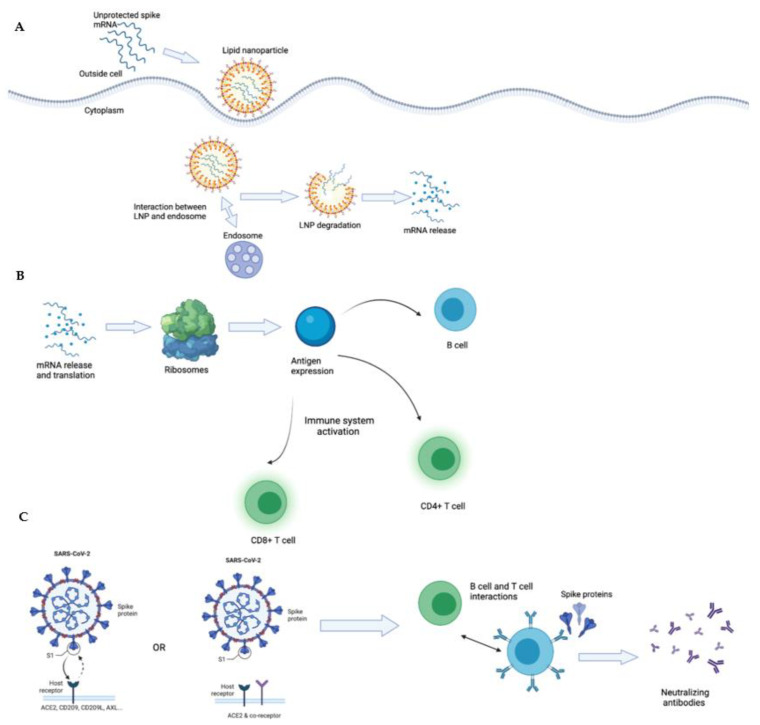
**Mechanism for lipid nanoparticles in mRNA Vaccine and activation of immune cells and immune response.** (**A**): This figure illustrates the mechanisms for the interaction between the lipid nanoparticles containing the spike mRNA, as well as mRNA release once in the body. (**B**): This figure illustrates the immune responses elicited once the mRNA spike proteins have entered the body, through the expressions of CD4+ and CD8+ T cells and B cells. (**C**): This figure details the infection of SARS-CoV-2 with the binding of the spike protein to the ACE2 receptor, ACE2 and co-receptor, and novel receptors to account for the potential methods of viral entry, and the neutralizing effects of B and T cell interactions after vaccination.

Unprotected mRNA can be encapsulated in a lipid nanoparticle to create a safe drug delivery system. Once the mRNA vaccine is injected, the positively charged lipid nanoparticle is attracted to the endosomes and this interaction starts the process of receptor-mediated endocytosis, which triggers the SLN to degrade and release the mRNA (Figure 2).

### 1.9. The Function and Potential Antagonist Risks of ACE2

Angiotensin-converting enzyme 2 (ACE2) is a metallopeptidase that counterpoises the angiotensin-converting enzyme (ACE) [129]. ACE2 is explicitly expressed in the digestive system and kidneys, but seldom in organs such as the lungs [15]. Subsequently, ACE2 has also been detected in bronchial and nasal epithelium [16].

ACE2 has been found to play a significant role in the pathology of COVID-19, since it serves as the functional receptor for COVID-19 entry [18]. Infection occurs when the COVID-19 S1 spike protein binds to ACE2 through the receptor binding domain (RBD) to tag the cell, which then allows for endocytosis [130]. This informs the immune system to recognize it as an infected cell, causing immune cells such as CD8+ and CD4+ T cells and B cells to kill it [131,132]. However, the expression of ACE2 is remarkably low in the respiratory tract and other human tissues, thereby suggesting there might be alternative or co-receptors [15].

### 1.10. Technological and Dosage Regimen of Current COVID-19 Vaccines

As of now, numerous COVID-19 vaccines have been developed and tested in many different populations, but it is still difficult to conduct a side-by-side comparison or predict the best vaccine because there is no long-term data available at present (Table 1). Generally speaking, the best vaccine should produce durable B and T cell responses without having any potential injury to normal tissue. The following table illustrates the major differences among some of the current major vaccines.

### 1.11. The Immune System and Immune Response to the COVID-19 mRNA Vaccines

Each individual’s immune system is unique because it is composed of innate immunity, which is adopted from parents, and acquired immunity, which is developed through interactions with the environment and exposure to different antigens [144]. Our immune systems also continuously change with age, and can be impacted by our lifestyles [145]. During the earlier stages of life, our immune systems are more robust and responsive to environmental changes and antigen stimulation; however, this responsiveness declines with age [146]. When an individual is exposed to an infectious agent or vaccine, a number of processes occur. First, antigen phagocytosis occurs by antigen presenting cells, which trigger the T cell response through the presented antigen, followed by cytokine release [147]. These steps are sequential, and proficient understanding of each step is crucial towards developing an effective vaccine.

With the Pfizer and Moderna mRNA vaccines, the lipid nanoparticles are injected into the deltoid. This muscle tissue consists of neurons, blood vessels, and muscle cells that contain T cells, antigen presenting cells through the role of cathepsins which present CD4 and CD8 cells, and natural killer cells [148,149]. The cells intake the lipid nanoparticles, which allows the COVID-19 spike protein to be synthesized in the cells. The COVID-19 spike protein can then be presented to CD4+ T cells once they are processed, and the cells then convert into memory T cells, and CD4 T cells aid B cells with the conversion into plasmocytes, which allows the production of antibodies [150,151].

Nevertheless, there are some adverse effects associated with the vaccines. For instance, the Moderna vaccine is more prone to induce side effects (e.g., fatigue and nausea, lymphadenopathy, erythema, swelling, fever, and joint pain) compared to Pfizer’s vaccine [68]. However, current data elucidates that both mRNA vaccines can produce a comparable humoral response, with minimal distinction in cellular immunity [68]. Nonetheless, phase 1 and 2 data shared by Pfizer reveals that their vaccine can generate a more powerful CD8 T-cell response compared to Moderna’s, which could prove helpful in fighting infection [37,152,153].

### 1.12. Concerns and Controversies Regarding Current COVID-19 Vaccines

Administration of the Oxford/AstraZeneca vaccine was previously suspended in eight European countries due to reports of rare blood clots. In Austria, one individual reportedly had multiple thrombosis and was reported dead 10 days after being vaccinated [154]. Moreover, a patient in Denmark died due to heparin-induced thrombocytopenia with an ischemic stroke which results from reactive antibodies that bind to platelet factor 4 and heparin, which may have been caused by an immune response to the vaccine [155]. Due to this, the European Medicine Agency reported that they were working with blood disorder professionals to investigate the thromboembolic reports [156]. Nonetheless, their further updates stated that the benefits derived from the vaccines outweighed the potential risks, and thereby recommend the general public to continue vaccinations since the reports of blood clots were not determined to be higher than expected in the general population.

In the US, the distribution of the Johnson & Johnson vaccine was temporarily paused due to reports of severe and rare blood clots in six individuals [157]. The blood clots appeared in unusual places such as the brain and abdomen and had characteristics such as low platelet count and fragmented cells that aid in blood coagulation, which are hallmarks of heparin-induced thrombocytopenia (HIT) [157]. Furthermore, the CDC conducted a study examining the reactions that occurred in a small subset of adults who received the Pfizer vaccine [152]. When examining serious adverse events, the vaccinated group had higher rates of serious side effects than the placebo group, which could be attributed to the vaccine. For example, seven vaccinated individuals had appendicitis, three had acute myocardial infarctions, and three had cerebrovascular incidents, compared to two, zero, and one participant(s) in the placebo groups, respectively [152]. The study included 2291 vaccinated and 2298 placebo individuals for the first dose, all aged between 18 and 55 years old [152]. There were also 1802 vaccinated and 1792 placebo individuals who were older than 55 years old [158]. While severe side effects are ultimately extremely rare and could be due to a specific batch of the vaccine or due to highly specific unknown circumstances regarding the individual who experienced these side effects, it is still crucial to investigate these reports and case studies to prevent future occurrences.

There have also been concerns regarding the data and trials for the COVID-19 vaccines. Although Oxford/AstraZeneca showed that their vaccine had a reported efficacy of 81.3%, there was some controversy regarding this claim, as there were two results from differing vaccination dosages (62% and 90%) which were combined, and led AstraZeneca to continue trials [71,159,160]. Furthermore, Gamaleya and Sinopharm have also faced controversies, with the Gamaleya clinical data having been disputed, and Sinopharm criticized for their scarcity of public data, which hinders the analysis of the efficacy or safety of the vaccine by professionals [89].

### 1.13. Currently Unknown Data for COVID-19 Vaccines

Although millions of people have been vaccinated worldwide, there is an absence of data comparing the B and T cell responses of all the vaccines [159,161,162]. It is very critical to conduct epitope mappings of antibodies generated from vaccines versus convalescence sera, since the processing and presentation of B or T cell epitopes could be different from that of naturally infected patients. As of now, there is also a lack of immune correlates for the protection against COVID-19, making the titre of neutralizing antibodies ambiguous [44]. Thus, large data sets are required to predict the protection of the vaccine, meaning that a significant amount of time is still required before a clear assessment of the efficacy of COVID-19 vaccines can be made.

### 1.14. Future Considerations for the COVID-19 Vaccines

Globally, there have been many variants of SARS-CoV-2 that have been emerging. Even just a year after initial infection, variants and cases of second COVID-19 infections have been reported; some originating in the UK, South Africa, and Brazil [163,164,165,166]. Given how common and problematic mutations are, two considerations must be made prior to implementing a treatment: avoid abuse of the therapeutic agent to prevent pressure mutation, and ensure the vaccine can prevent immune escape by the virus. The viral mutations and variants have specific attributes resulting in increases of infections to a specific variant, changes in transmission, diagnosis and therapeutic treatment, and changes in the elicited immune cell populations [167]. It is crucial to consider mutations when determining which vaccine to administer or receive, as certain vaccines have shown reduced efficacy against certain COVID-19 variants. These new variants have been classified into three groups, which include variant of interest (VOI), variant of concern (VOC), and variant of high consequence (VOHC) [168]. Possible attributes within the VOI include increased transmission, decreased susceptibility to monoclonal antibodies (mAbs), and resistance to neutralization by convalescent and post-vaccination sera [168]. The variants of interest that have been monitored by the CDC include Epsilon (B.1.427 and B.1429), Eta (B.1.525) Iota (B.1.526), and Kappa (B.1.617.1). The characteristics of the VOC include a 50% increase in transmission, increased severity in the infections, reduced effectiveness of treatments, and reduction in therapies [168]. The VOC that are prevalent worldwide include Alpha (B.1.1.7), Beta (B.1.351), Delta (B.1.617.2), and Gamma (P.1). During the second and third waves of infection from SARS-CoV-2 resulting from the Alpha and Beta variants, data on the effectiveness of the vaccinations and characteristics of the variants were collected. Against the documented infections of the Alpha variant, the effectiveness of the Pfizer-BioNTech vaccine was 85.5% and for the Beta variant, the effectiveness was 75.0% [169]. These findings support the claim that the vaccine is effective against the variants, however, the Beta variant resulted in an effectiveness that was about 20% below the reported 90% effectiveness [169]. Moreover, preliminary efficacy data from phase 3 clinical trials released by Johnson & Johnson and Novavax have shown that these vaccines are less effective against the Beta variant, while still being effective against other strains [170]. Furthermore, the Oxford/AstraZeneca vaccine was even suspended in South Africa for its ineffectiveness against the Beta variant [170]. The most prominent variant that has resulted in many new infections worldwide is the Delta variant, which first appeared in India late 2020 [171]. The Delta variant has shown to spread faster compared to other variants and is resistant to mAbs, including Bamlanivimab, Casirivimab, Etesevimab, and Imdevimab [172]. The potency of the mAbs were measured to determine its efficacy against the Delta variant and it was found to be less efficient when compared to other variants. Additionally, the Delta variant reduces neutralization, as there is a loss of antibodies binding [172]. The Delta variant has also produced many breakthrough infections similar to unvaccinated people but the infection time for individuals who are fully vaccinated decreases, compared to those who are unvaccinated [173]. In individuals who have received two doses of the Pfizer-BioNTech vaccine, the effectiveness of the vaccine is 88% and decreases to 78% effectiveness after 90 days of receiving the vaccine [174,175]. Moreover, the Oxford/AstraZeneca vaccine has been found to be 67% effective against the Delta variant [174,175]. As of now, there are no SARS-CoV-2 variants that have been classified as a VOHC, which arises from strong evidence in the reduction of vaccine effectiveness, severe infections, and reduced susceptibility to many of the Emergency Use Authorization therapeutics [168].

Furthermore, the virus seems to affect men and women differently, with men often displaying higher hospitalization rates [20,176,177]. The COVID-19 bias also extends to a greater male fatality being reported in 37 out of the 38 countries that have released their sex-disaggregated data [178]. However, it is not currently known if this is due to immune system genes encoded in the X chromosome, or if sex hormones play a considerable role in the COVID-19 immune response [179]. The disproportionate cases of COVID-related hospitalizations may also be attributed to the higher prevalence of chronic illnesses (i.e., cardiovascular diseases) or the increased ACE2 level in circulation among men compared to women [177]. Nevertheless, this trend of higher male mortality was also noted with the previous SARS-CoV, as well as MERS-CoV [178].

## 2. Conclusions

Ultimately, time will be the main factor in determining which prophylactic vaccine will be most effective in slowing the spread of COVID-19. As more data becomes available, the efficacy of the vaccines will continue to be analyzed and more knowledge will become available as the pandemic progresses. Currently, the adjuvant, the method of vaccination, the age of vaccine recipients, and the degree of pre-existing immunity should all be considered when designing a safe vaccine strategy. Furthermore, vaccines that exclude ineffectively neutralizing epitopes and include epitopes that are recognized for their protective immune responses could theoretically decrease the chance of ADE [44]. For example, traditional adjuvant-based vaccines could be effectively implemented, since they are well-documented and have been utilized for over a century. Additionally, the significant progress in recombinant technology has allowed for the facile, mass production of antigens, as well as the modification of different expression systems, such as mammalian cell lines [180]. Peptide-based vaccines may also allow for the selection of the adjuvant that could be employed to best modulate the immune system as desired, to avoid potential unwanted responses [181]. This could be utilized as a preferred alternative to the current mRNA, viral vector, and inactive whole virus vaccines, depending on future data.

## Figures and Tables

**Table 1:** **Comparison of different vaccines against COVID-19.** This chart details the exposure method, required doses, technological overview, and manufacturers of current COVID-19 vaccines.

Company	Required Doses	Technological and Dosage Overview
Exposure method: RNA (mRNA)		
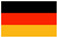 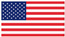 Pfizer & BioNTech	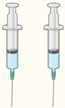	The Pfizer BioNTech vaccine dosage is 0.3 mL and requires 2 doses, 21 days apart and the Moderna vaccine dosage is 0.5 mL and requires 2 doses, 20 days apart [133,134].Lipid nanoparticles encapsulate mRNA, allowing for the precise delivery of the genetic components of the vaccine, optimizing the translation of the proteins [135].
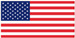 Moderna	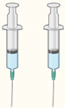	
Exposure method: Viral Vector		
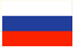 Sputnik V (recombinant adenovirus type 26 and 5)	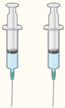	The SputnikV vaccine dosage is 0.5 mL with 2 vaccine doses, 21 days apart [136]. The Johnson & Johnson vaccine dosage is 0.5 mL and requires 1 vaccine dose [137]. The Oxford AstraZeneca vaccine dosage is 0.5 mL with 2 vaccine doses, 8–12 weeks apart [138].Utilizes an adenovirus vector to elicit spike proteins on cell surfaces resulting in immune responses through the activation of antibodies [75].
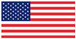 Johnson & Johnson (recombinant adenovirus type 26 vector)	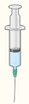	
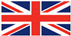 Oxford AstraZeneca (adenovirus)	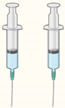	
Exposure method: Protein based		
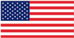 Novavax	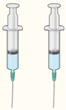	Utilizes the S protein from SARS-CoV-2 with recombinant protein nanoparticles and adjuvant MatrixM to elicit desired immune responses, and requires 2 vaccine doses, 21 days apart [139].
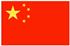 Zifivax	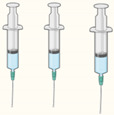	The vaccine is composed of antigens and viral particles of SARS-CoV-2 that will generate an immune response and requires 3 vaccine doses, 0.5 mL each, over the course of 2 months [140,141].
Exposure method: Peptide-antigen based		
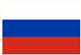 EpiVacCorona	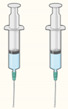	Three peptide antigens for SARS-CoV-2 are synthesized consisting of the spike protein and a chimeric protein, an aluminum hydroxide adjuvant is utilized to synthesize the vaccine, and requires 2 vaccine doses, 0.5 mL each, over the course of 21–28 days [142].
Exposure Method: Inactivated Virus		
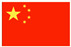 WIBP-CorV Sinopharm	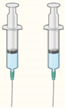	Inactive whole virus technology has been widely studied and is effective for individuals with impaired immune systems and requires 2 vaccine doses, 0.5 mL each, with an interval of 3–4 weeks [132,143].
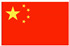 BBIBP-CorV Sinopharm	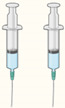	Inactive whole virus technology has been widely studied and is effective for individuals with impaired immune systems and requires 2 vaccine doses, 0.5 mL each, with an interval of 3–4 weeks [132,143].

## Data Availability

Not applicable.

## Abbreviations

ACE2: Angiotensin-converting enzyme 2; ACE: Angiotensin-converting enzyme; ADE: Antibody dependent enhancement; HIT: Heparin-induced thrombocytopenia; HIV: Human immunodeficiency virus; IM: Intramuscular; J&J: Johnson & Johnson; MERS-CoV: Middle Eastern Respiratory Syndrome Coronavirus; mAbs: monoclonal antibody; PEGylated lipid: polyethylene glycol lipid; PD: Pharmacodynamic; PK: Pharmacokinetic; RBD: Receptor binding domain; SARS-CoV: Severe acute respiratory syndrome coronavirus; SARS-CoV-2: Severe acute respiratory syndrome coronavirus 2; Sf9: *Spodoptera frugiperda*; SLN, sLNPs: Solid lipid nanoparticles; S: Spike; VOC: Variant of concern; VOHC: Variant of high consequence; VOI: Variant of interest.

## References

[1] Singhal T. (2020). A Review of Coronavirus Disease-2019 (COVID-19). Indian J. Pediatr..

[2] John Hopkins University of Medicine (2021). Coronavirus Resource Center. https://coronavirus.jhu.edu/.

[3] Villa S., Lombardi A., Mangioni D., Bozzi G., Bandera A., Gori A., Raviglione M. (2020). The COVID-19 pandemic preparedness or lack thereof: From China to Italy. Glob. Health Med..

[4] Cheng C., Zhang T., Song C., Shen S., Jiang Y., Zhang X. (2020). The Coupled Impact of Emergency Responses and Population Flows on the COVID-19 Pandemic in China. Geohealth.

[5] Wang Y., Li B., Gouripeddi R., Facelli J.C. (2021). Human activity pattern implications for modeling SARS-CoV-2 transmission. Comput. Methods Programs Biomed..

[6] Buchanan N.D., Aslaner D.M., Adelstein J., MacKenzie D.M., Wold L.E., Gorr M.W. (2021). Remote Work during the COVID-19 Pandemic: Making the Best of It. Physiology.

[7] Freppel W., Merindol N., Rallu F., Bergevin M. (2020). Efficient SARS-CoV-2 detection in unextracted oro-nasopharyngeal specimens by rRT-PCR with the Seegene Allplex™ 2019-nCoV assay. Virol. J..

[8] Bolcato M., Aurilio M., Aprile A., Di Mizio G., Della Pietra B., Feola A. (2021). Take-Home Messages from the COVID-19 Pandemic: Strengths and Pitfalls of the Italian National Health Service from a Medico-Legal Point of View. Healthcare.

[9] Bolcato M., Rodriguez D., Feola A., Di Mizio G., Bonsignore A., Ciliberti R., Tettamanti C., Aurillo M., Aprile A. (2021). COVID-19 Pandemic and Equal Access to Vaccines. Vaccines.

[10] WHO (2021). Tracking SARS-CoV-2 Variants.

[11] Chams N., Chams S., Badran R., Shams A., Araji A., Raad M., Mukhopadhyay S., Stroberg E., Duval E., Barton L. (2020). COVID-19: A Multidisciplinary Review. Front. Public Health.

[12] Hu B., Guo H., Zhou P., Shi Z.L. (2021). Characteristics of SARS-CoV-2 and COVID-19. Nat. Rev. Microbiol..

[13] Schoeman D., Fielding B.C. (2019). Coronavirus envelope protein: Current knowledge. Virol. J..

[14] Puelles V.G., Lütgehetmann M., Lindenmeyer M.T., Sperhake J.P., Wong M.N., Allweiss L., Chilla S., Heineman A., Wanner N., Liu S. (2020). Multiorgan and Renal Tropism of SARS-CoV-2. N. Engl. J. Med..

[15] Wang S., Qiu Z., Hou Y., Deng X., Xu W., Zheng T., Wu P., Xie S., Bian W., Zhang C. (2021). AXL is a candidate receptor for SARS-CoV-2 that promotes infection of pulmonary and bronchial epithelial cells. Cell Res..

[16] Lukassen S., Chua R.L., Trefzer T., Kahn N.C., Schneider M.A., Muley T., Winter H., Meister M., Veith C., Boots A. (2020). SARS-CoV-2 receptor ACE2 and TMPRSS2 are primarily expressed in bronchial transient secretory cells. EMBO J..

[17] Amraei R., Yin W., Napoleon M.A., Suder E.L., Berrigan J., Zhao Q., Olejnik J., Chandler K., Xia C., Feldman J. (2021). CD209L/L-SIGN and CD209/DC-SIGN act as receptors for SARS-CoV-2. bioRxiv.

[18] Cuervo N.Z., Grandvaux N. (2020). ACE2: Evidence of role as entry receptor for SARS-CoV-2 and implications in comorbidities. Elife.

[19] Edwards D., Hickey A., Batycky R., Griel L., Lipp M., Dehaan W., Clarke R., Hava D., Perry J., Laurenzi B. (2020). A New Natural Defense Against Airborne Pathogens. QRB Discov..

[20] Chen Z., Wherry E.J. (2020). T cell responses in patients with COVID-19. Nat. Rev. Immunol..

[21] Golubev A.G. (2020). COVID-19: A Challenge to Physiology of Aging. Front. Physiol..

[22] Wang B., Li R., Lu Z., Huang Y. (2020). Does comorbidity increase the risk of patients with COVID-19: Evidence from meta-analysis. Aging.

[23] Harapan H., Itoh N., Yufika A., Winardi W., Keam S., Te H., Megawati D., Hayati Z., Wagner A., Mudatsir M. (2020). Coronavirus disease 2019 (COVID-19): A literature review. J. Infect. Public Health..

[24] Hazlett C., Wulf D.A., Pasaniuc B., Arah O.A., Erlandson K.M., Montague B.T. (2020). Credible learning of hydroxychloroquine and dexamethasone effects on COVID-19 mortality outside of randomized trials. medRxiv.

[25] Singh A.K., Majumdar S., Singh R., Misra A. (2020). Role of corticosteroid in the management of COVID-19: A systemic review and a Clinician’s perspective. Diabetes Metab. Syndr. Clin. Res. Rev..

[26] Hossen M.S., Barek M.A., Jahan N., Safiqul Islam M. (2020). A Review on Current Repurposing Drugs for the Treatment of COVID-19: Reality and Challenges. SN Compr. Clin. Med..

[27] Corey L., Mascola J.R., Fauci A.S., Collins F.S. (2020). A strategic approach to COVID-19 vaccine R&D. Science.

[28] Chowdhury F.R., Hoque A., Chowdhury F.U.H., Amin M.R., Rahim A., Rahman M.M., Yasmin R., Amin M.R., Miah M.T., Kalam M.A. (2020). Convalescent plasma transfusion therapy in severe COVID-19 patients- a safety, efficacy and dose response study: A structured summary of a study protocol of a phase II randomized controlled trial. Trials.

[29] Lee W.T., Goo T.T., Lim W.W., Toh H.C., Yasai Y. (2020). Hospital Seeing More Personal Mobility Device Accidents and Serious Injuries Despite Active Mobility Act. J. Emerg. Trauma Shock.

[30] Maor Y., Cohen D., Paran N., Israely T., Ezra V., Axelrod O., Shinar E., Izak M., Rahav G., Rahimi-Levene N. (2020). Compassionate use of convalescent plasma for treatment of moderate and severe pneumonia in COVID-19 patients and association with IgG antibody levels in donated plasma. EClinicalMedicine.

[31] Liu S.T.H., Lin H.M., Baine I., Wajnberg A., Gumprecht J.P., Rahman F., Rodriguez D., Tandon P., assily-Marcus A., Bander J. (2020). Convalescent plasma treatment of severe COVID-19: A propensity score-matched control study. Nat. Med..

[32] Duan K., Liu B., Li C., Zhang H., Yu T., Qu J., Zhou M., Chen L., Meng S., Hu Y. (2020). Effectiveness of convalescent plasma therapy in severe COVID-19 patients. Proc. Natl. Acad. Sci. USA.

[33] Hinman A. (1999). Eradication of vaccine-preventable diseases. Annu. Rev. Public Health.

[34] Silveira M.M., Moreira G.M.S.G., Mendonça M. (2021). DNA vaccines against COVID-19: Perspectives and challenges. Life Sci..

[35] Anderson E.J., Rouphael N.G., Widge A.T., Jackson L.A., Roberts P.C., Makhene M., Chappell J.D., Denison M.R., Stevens L.J., Pruijssers A.J. (2020). Safety and Immunogenicity of SARS-CoV-2 mRNA-1273 Vaccine in Older Adults. N. Engl. J. Med..

[36] Lu J., Lu G., Tan S., Xia J., Xiong H., Yu X., Qi Q., Yu X., Li L., Yu H. (2020). A COVID-19 mRNA vaccine encoding SARS-CoV-2 virus-like particles induces a strong antiviral-like immune response in mice. Cell Res..

[37] Jackson L.A., Anderson E.J., Rouphael N.G., Roberts P.C., Makhene M., Coler R.N., McCullough M.P., Chappell J.D., Denison M.R., Stevens L.J. (2020). An mRNA Vaccine against SARS-CoV-2—Preliminary Report. N. Engl. J. Med..

[38] Kalita P., Padhi A.K., Zhang K.Y.J., Tripathi T. (2020). Design of a peptide-based subunit vaccine against novel coronavirus SARS-CoV-2. Microb. Pathog..

[39] Abbasi J. (2020). COVID-19 and mRNA Vaccines—First Large Test for a New Approach. JAMA.

[40] Huang Q., Zeng J., Yan J. (2021). COVID-19 mRNA vaccines. J. Genet. Genom..

[41] Pardi N., Hogan M.J., Porter F.W., Weissman D. (2018). mRNA vaccines—A new era in vaccinology. Nat. Rev. Drug Discov..

[42] Chowdhury M.A., Hossain N., Kashem M.A., Shahid M.A., Alam A. (2020). Immune response in COVID-19: A review. J. Infect. Public Health.

[43] Nakanaga K., Yamanouchi K., Fujiwara K. (1986). Protective effect of monoclonal antibodies on lethal mouse hepatitis virus infection in mice. J. Virol..

[44] Jeyanathan M., Afkhami S., Smaill F., Miller M.S., Lichty B.D., Xing Z. (2020). Immunological considerations for COVID-19 vaccine strategies. Nat. Rev. Immunol..

[45] Coughlin M., Lou G., Martinez O., Masterman S.K., Olsen O.A., Moksa A.A., Farzan M., Babcook J.S., Prabhakar B.S. (2007). Generation and characterization of human monoclonal neutralizing antibodies with distinct binding and sequence features against SARS coronavirus using XenoMouse^®^. Virology.

[46] To K.K., Tsang O.T., Leung W.S., Tam A.R., Wu T.C., Lung D.C., Yip C.C.-Y., Cai J.-P., Chan J.M.-C., Chik T.S.-H. (2020). Temporal profiles of viral load in posterior oropharyngeal saliva samples and serum antibody responses during infection by SARS-CoV-2: An observational cohort study. Lancet Infect. Dis..

[47] Liu W., Liu L., Kou G., Zheng Y., Ding Y., Ni W., Wang Q., Tan L., Wu W., Tang S. (2020). Evaluation of Nucleocapsid and Spike Protein-Based Enzyme-Linked Immunosorbent Assays for Detecting Antibodies against SARS-CoV-2. J. Clin. Microbiol..

[48] Long Q.X., Liu B.Z., Deng H.J., Wu G.C., Deng K., Chen Y.K., Liao P., Qui J.-F., Lin Y., Cai X.-F. (2020). Antibody responses to SARS-CoV-2 in patients with COVID-19. Nat. Med..

[49] Walls A.C., Park Y.J., Tortorici M.A., Wall A., McGuire A.T., Veesler D. (2020). Structure, Function, and Antigenicity of the SARS-CoV-2 Spike Glycoprotein. Cell.

[50] Jiang S., Hillyer C., Du L. (2020). Neutralizing Antibodies against SARS-CoV-2 and Other Human Coronaviruses. Trends Immunol..

[51] Duan J., Yan X., Guo X., Cao W., Han W., Qi C., Feng J., Yang D., Gao G., Jin G. (2005). A human SARS-CoV neutralizing antibody against epitope on S2 protein. Biochem. Biophys. Res. Commun..

[52] Mueller A.L., McNamara M.S., Sinclair D.A. (2020). Why does COVID-19 disproportionately affect older people?. Aging.

[53] Nanda A., Vura N.V.R.K., Gravenstein S. (2020). COVID-19 in older adults. Aging Clin. Exp. Res..

[54] Kuri-Cervantes L., Pampena M.B., Meng W., Rosenfeld A.M., Ittner C.A.G., Weisman A.R., Agyekum R.S., Mathew D., Baxter A.E., Vella L.A. (2020). Comprehensive mapping of immune perturbations associated with severe COVID-19. Sci. Immunol..

[55] Giamarellos-Bourboulis E.J., Netea M.G., Rovina N., Akinosoglou K., Antoniadou A., Antonakos N., Damoraki G., Gkavogianni T., Adami M.-E., Katsaounou P. (2020). Complex Immune Dysregulation in COVID-19 Patients with Severe Respiratory Failure. Cell Host Microbe.

[56] Merad M., Martin J.C. (2020). Author Correction: Pathological inflammation in patients with COVID-19: A key role for monocytes and macrophages. Nat. Rev. Immunol..

[57] Grifoni A., Weiskopf D., Ramirez S.I., Mateus J., Dan J.M., Moderbacher C.R., Rawlings S.A., Sutherland A., Premkumar L., Jadi R.S. (2020). Targets of T Cell Responses to SARS-CoV-2 Coronavirus in Humans with COVID-19 Disease and Unexposed Individuals. Cell.

[58] Braun J., Loyal L., Frentsch M., Wendisch D., Georg P., Kurth F., Hippenstiel S., Dingeldey M., Kruse B., Fauchere F. (2020). SARS-CoV-2-reactive T cells in healthy donors and patients with COVID-19. Nature.

[59] Sariol A., Perlman S. (2020). Lessons for COVID-19 Immunity from Other Coronavirus Infections. Immunity.

[60] Clem A.S. (2011). Fundamentals of vaccine immunology. J. Glob. Infect. Dis..

[61] Huang Y., Yang C., Xu X.F., Xu W., Liu S.W. (2020). Structural and functional properties of SARS-CoV-2 spike protein: Potential antivirus drug development for COVID-19. Acta Pharmacol. Sin..

[62] Saponaro F., Rutigliano G., Sestito S., Bandini L., Storti B., Bizzarri R., Zucchi R. (2020). ACE2 in the Era of SARS-CoV-2: Controversies and Novel Perspectives. Front. Mol. Biosci..

[63] Chagla Z. (2021). The BNT162b2 (BioNTech/Pfizer) vaccine had 95% efficacy against COVID-19 ≥7 days after the 2nd dose. Ann. Intern. Med..

[64] Walsh E.E., Frenck R.W., Falsey A.R., Kitchin N., Absalon J., Gurtman A., Lockhart S., Neuzil K., Mulligan M.J., Bailey R. (2020). Safety and Immunogenicity of Two RNA-Based Covid-19 Vaccine Candidates. N. Engl. J. Med..

[65] Cohen J. (2020). Vaccine wagers on coronavirus surface protein pay off. Science.

[66] Gaebler C., Nussenzweig M.C. (2020). All eyes on a hurdle race for a SARS-CoV-2 vaccine. Nature.

[67] Brussow H. (2021). COVID-19: Vaccination Problems. Environ. Microbiol..

[68] Meo S.A., Bukhari I.A., Akram J., Meo A.S., Klonoff D.C. (2021). COVID-19 vaccines: Comparison of biological, pharmacological characteristics and adverse effects of Pfizer/BioNTech and Moderna Vaccines. Eur. Rev. Med. Pharmacol. Sci..

[69] Pacheco T.J.A., da Silva V.C.M., de Souza D.G. (2020). Nano COVID-19 Vaccines: The firsts RNA lipid nanoparticle vaccines being approved from history—Review. Res. Soc. Dev..

[70] Knoll M.D., Wonodi C. (2021). Oxford-AstraZeneca COVID-19 vaccine efficacy. Lancet.

[71] Mahase E. (2020). Covid-19: Oxford vaccine is up to 90% effective, interim analysis indicates. BMJ.

[72] Arashkia A., Jalilvand S., Mohajel N., Afchangi A., Azadmanesh K., Salehi-Vaziri M., Fazlalipour M., Pouriayevali M.H., Jalali T., Nasab S.D.M. (2020). Severe acute respiratory syndrome-coronavirus-2 spike (S) protein based vaccine candidates: State of the art and future prospects. Rev. Med. Virol..

[73] Ewer K.J., Barrett J.R., Belij-Rammerstorfer S., Sharpe H., Makinson R., Morter R., Flaxman D.W., Bellamy D., Bittaye M., Dold C. (2021). T cell and antibody responses induced by a single dose of ChAdOx1 nCoV-19 (AZD1222) vaccine in a phase 1/2 clinical trial. Nat. Med..

[74] Jones I., Roy P. (2021). Sputnik V COVID-19 vaccine candidate appears safe and effective. Lancet.

[75] Prevention CfDCa (2021). Understanding Viral Vector COVID-19 Vaccines. CDC. https://www.cdc.gov/coronavirus/2019-ncov/vaccines/different-vaccines/viralvector.html.

[76] Ricke D.O. (2021). Two Different Antibody-Dependent Enhancement (ADE) Risks for SARS-CoV-2 Antibodies. Front. Immunol..

[77] Administration UFD (2021). FDA Issues Emergency Use Authorization for Third COVID-19 Vaccine. https://www.fda.gov/news-events/press-announcements/fda-issues-emergency-use-authorization-third-covid-19-vaccine.

[78] Burki T.K. (2020). The Russian vaccine for COVID-19. Lancet Respir. Med..

[79] Balakrishnan V.S. (2020). The arrival of Sputnik, V. Lancet Infect. Dis..

[80] Mahase E. (2021). Covid-19: Russian vaccine efficacy is 91.6%, show phase III trial results. BMJ.

[81] Voysey M., Clemens S.A.C., Madhi S.A., Weckx L.Y., Folegatti P.M., Aley P.K., Angus B., Baillie V.L., Barnabas S.L., Bhorat Q.E. (2021). Safety and efficacy of the ChAdOx1 nCoV-19 vaccine (AZD1222) against SARS-CoV-2: An interim analysis of four randomised controlled trials in Brazil, South Africa, and the UK. Lancet.

[82] Sofia Mouthinho M.W. (2021). Is Russia’s COVID-19 Vaccine Safe?. Brazil’s Veto of Sputnik V Sparks Lawsuit Threat and Confusion Science..

[83] Johnson J. (2020). A Randomized, Double-Blind, Placebo-Controlled Phase 3 Study to Assess the Efficacy and Safety of Ad26.COV2.S for the Prevention of SARS-CoV-2-Mediated COVID-19 in Adults Aged 18 Years and Older. https://www.jnj.com/coronavirus/ensemble-1-study-protocol.

[84] Brüssow H. (2020). Efforts towards a COVID-19 vaccine. Environ. Microbiol..

[85] Mercado N.B., Zahn R., Wegmann F., Loos C., Chandrashekar A., Yu J., Liu J., Peter L., McMahan K., Tostanoski L.H. (2020). Single-shot Ad26 vaccine protects against SARS-CoV-2 in rhesus macaques. Nature.

[86] Lavigne S.E. (2021). Vaccine hesitancy: Root causes and possible solutions. Can. J. Dent. Hyg..

[87] Kyriakidis N.C., López-Cortés A., González E.V., Grimaldos A.B., Prado E.O. (2021). SARS-CoV-2 vaccines strategies: A comprehensive review of phase 3 candidates. NPJ Vaccines.

[88] Xia S., Zhang Y., Wang Y., Wang H., Yang Y., Gao G.F., Tan W., Wu G., Xu M., Lou Z. (2021). Safety and immunogenicity of an inactivated SARS-CoV-2 vaccine, BBIBP-CorV: A randomised, double blind, placebo-controlled, phase 1/2 trial. Lancet Infect. Dis..

[89] Baraniuk C. (2021). What do we know about China’s covid-19 vaccines?. BMJ.

[90] Hotez P.J., Nuzhath T., Callaghan T., Colwell B. (2021). COVID-19 Vaccine Decisions: Considering the Choices and Opportunities. Microbes Infect..

[91] Al Kaabi N., Zhang Y., Xia S., Yang Y., Al Qahtani M.M., Abdulrazzaq N., Nusair M.A., Hassany M., Jawad J.S., Abdalla J. (2021). Effect of 2 Inactivated SARS-CoV-2 Vaccines on Symptomatic COVID-19 Infection in Adults: A Randomized Clinical Trial. JAMA.

[92] Mallapaty S. (2021). China’s COVID vaccines are going global—But questions remain. Nature.

[93] Keech C., Albert G., Cho I., Robertson A., Reed P., Neal S., Plested J.S., Zhu M., Cloney-Clark S., Zhou H. (2020). Phase 1–2 Trial of a SARS-CoV-2 Recombinant Spike Protein Nanoparticle Vaccine. N. Engl. J. Med..

[94] Bangaru S., Ozorowski G., Turner H.L., Antanasijevic A., Huang D., Wang X., Torres J., Diedrich J.K., Tian J.-H., Portnoff A.D. (2020). Structural analysis of full-length SARS-CoV-2 spike protein from an advanced vaccine candidate. Science.

[95] Novavax (2021). Recombinant Nanoparticle Vaccine Technology. https://www.novavax.com/our-unique-technology.

[96] Chung Y.H., Beiss V., Fiering S.N., Steinmetz N.F. (2020). COVID-19 Vaccine Frontrunners and Their Nanotechnology Design. ACS Nano.

[97] Callaway E., Mallapaty S. (2021). Novavax offers first evidence that COVID vaccines protect people against variants. Nat. Cell Biol..

[98] Yang S., Li Y., Dai L., Wang J., He P., Li C., Fang X., Wang C., Zhao X., Huang E. (2021). Safety and immunogenicity of a recombinant tandem-repeat dimeric RBD-based protein subunit vaccine (ZF2001) against COVID-19 in adults: Two randomised, double-blind, placebo-controlled, phase 1 and 2 trials. Lancet Infect. Dis..

[99] Baraniuk C. (2021). Covid-19: What do we know about Sputnik V and other Russian vaccines?. BMJ.

[100] Flanagan K.L., MacIntyre C.R., McIntyre P.B., Nelson M.R. (2021). SARS-CoV-2 Vaccines: Where Are We Now?. J. Allergy Clin. Immunol. Pract..

[101] Naseri N., Valizadeh H., Zakeri-Milani P. (2015). Solid Lipid Nanoparticles and Nanostructured Lipid Carriers: Structure, Preparation and Application. Adv. Pharm. Bull..

[102] García-Pinel B., Porras-Alcalá C., Ortega-Rodríguez A., Sarabia F., Prados J., Melguizo C., Lopez-Romero J.M. (2019). Lipid-Based Nanoparticles: Application and Recent Advances in Cancer Treatment. Nanomaterials.

[103] Guevara M.L., Persano F., Persano S. (2020). Advances in Lipid Nanoparticles for mRNA-Based Cancer Immunotherapy. Front. Chem..

[104] Chatzikleanthous D., O’Hagan D.T., Adamo R. (2021). Lipid-Based Nanoparticles for Delivery of Vaccine Adjuvants and Antigens: Toward Multicomponent Vaccines. Mol. Pharm.

[105] Przybytkowski E., Behrendt M., Dubois D., Maysinger D. (2009). Nanoparticles can induce changes in the intracellular metabolism of lipids without compromising cellular viability. FEBS J..

[106] Reichmuth A.M., Oberli M.A., Jaklenec A., Langer R., Blankschtein D. (2016). mRNA vaccine delivery using lipid nanoparticles. Ther. Deliv..

[107] Moss K.H., Popova P., Hadrup S.R., Astakhova K., Taskova M. (2019). Lipid Nanoparticles for Delivery of Therapeutic RNA Oligonucleotides. Mol. Pharm..

[108] Mehnert W., Mäder K. (2001). Solid lipid nanoparticles: Production, characterization and applications. Adv. Drug Deliv. Rev..

[109] Polack F.P., Thomas S.J., Kitchin N., Absalon J., Gurtman A., Lockhart S., Perez J.L., Marc G.P., Moreira E.D., Zerbini C. (2020). Safety and Efficacy of the BNT162b2 mRNA Covid-19 Vaccine. N. Engl. J. Med..

[110] (2020). Asteroid treasure, COVID vaccine and public peer review. Nature.

[111] Eygeris Y., Patel S., Jozic A., Sahay G. (2020). Deconvoluting Lipid Nanoparticle Structure for Messenger RNA Delivery. Nano Lett..

[112] Buschmann M.D., Carrasco M.J., Alishetty S., Paige M., Alameh M.G., Weissman D. (2021). Nanomaterial Delivery Systems for mRNA Vaccines. Vaccines.

[113] (2021). Messengers of hope. Nat. Biotechnol..

[114] Park K.S., Sun X., Aikins M.E., Moon J.J. (2021). Non-viral COVID-19 vaccine delivery systems. Adv. Drug Deliv. Rev..

[115] Mocan T., Matea C.T., Iancu C., Agoston-Coldea L., Mocan L., Orasan R. (2016). Hypersensitivity and nanoparticles: Update and research trends. Clujul. Med.

[116] Moghimi S.M. (2021). Allergic Reactions and Anaphylaxis to LNP-Based COVID-19 Vaccines. Mol. Ther..

[117] Sellaturay P., Nasser S., Islam S., Gurugama P., Ewan P.W. (2021). Polyethylene glycol (PEG) is a cause of anaphylaxis to the Pfizer/BioNTech mRNA COVID-19 vaccine. Clin. Exp. Allergy.

[118] Mukherjee S., Ray S., Thakur R.S. (2009). Solid lipid nanoparticles: A modern formulation approach in drug delivery system. Indian J. Pharm. Sci..

[119] Cataldi M., Vigliotti C., Mosca T., Cammarota M., Capone D. (2017). Emerging Role of the Spleen in the Pharmacokinetics of Monoclonal Antibodies, Nanoparticles and Exosomes. Int. J. Mol. Sci..

[120] Czajkowska-Kośnik A., Szekalska M., Winnicka K. (2019). Nanostructured lipid carriers: A potential use for skin drug delivery systems. Pharmacol. Rep..

[121] Patel D.K., Kesharwani R., Kumar V. (2019). Lipid Nanoparticle Topical and Transdermal Delivery: A Review on Production, Penetration Mechanism to Skin. Int. J. Pharm. Investig..

[122] Hassett K.J., Benenato K.E., Jacquinet E., Lee A., Woods A., Yuzhakov O., Himansu S., Deterling J., Geilich B.M., Ketova T. (2019). Optimization of Lipid Nanoparticles for Intramuscular Administration of mRNA Vaccines. Mol. Ther. Nucleic Acids.

[123] Meng C., Chen Z., Li G., Welte T., Shen H. (2021). Nanoparticles for mRNA Therapeutics. Adv. Therap..

[124] Wang R., Luo X., Liu F., Luo S. (2021). Confronting the threat of SARS-CoV-2: Realities, challenges and therapeutic strategies (Review). Exp. Ther. Med..

[125] Hrkach J., Langer R. (2020). From micro to nano: Evolution and impact of drug delivery in treating disease. Drug. Deliv. Transl. Res..

[126] Prub B.M. (2021). Current State of the First COVID-19 Vaccines. Vaccines.

[127] Bettini E., Locci M. (2021). SARS-CoV-2 mRNA Vaccines: Immunological Mechanism and Beyond. Vaccines.

[128] Speiser D.E., Bachmann M.F. (2020). COVID-19: Mechanisms of Vaccination and Immunity. Vaccines.

[129] Turner A.J., Hiscox J.A., Hooper N.M. (2004). ACE2: From vasopeptidase to SARS virus receptor. Trends Pharm. Sci..

[130] Shang J., Wan Y., Luo C., Ye G., Geng Q., Auerbach A., Li F. (2020). Cell entry mechanisms of SARS-CoV-2. Proc. Natl. Acad. Sci. USA.

[131] Wang H., Yang P., Liu K., Guo F., Zhang Y., Zhang G., Jiang C. (2008). SARS coronavirus entry into host cells through a novel clathrin- and caveolae-independent endocytic pathway. Cell Res..

[132] Sadarangani M., Marchant A., Kollmann T.R. (2021). Immunological mechanisms of vaccine-induced protection against COVID-19 in humans. Nat. Rev. Immunol..

[133] Prevention CfDCa (2021). Moderna CDC. https://www.cdc.gov/coronavirus/2019-ncov/vaccines/different-vaccines/Moderna.html.

[134] Prevention CfDCa (2021). Pfizer-BioNTech COVID-19 Overview and Safety: CDC. https://www.cdc.gov/coronavirus/2019-ncov/vaccines/different-vaccines/Pfizer-BioNTech.html.

[135] Sahin U., Muik A., Derhovanessian E., Vogler I., Kranz L.M., Vormehr M., Baum A., Pascal K., Quandt J., Maurus D. (2020). COVID-19 vaccine BNT162b1 elicits human antibody and T. Nature.

[136] Nogrady B. (2021). Mounting evidence suggests Sputnik COVID vaccine is safe and effective. Nat. Cell Biol..

[137] CDC (2021). Janssen COVID-19 Vaccine (Johnson & Johnson). https://www.cdc.gov/vaccines/covid-19/info-by-product/janssen/index.html.

[138] WHO (2021). The Oxford/AstraZeneca COVID-19 Vaccine: What You Need to Know. https://www.who.int/news-room/feature-stories/detail/the-oxford-astrazeneca-covid-19-vaccine-what-you-need-to-know.

[139] Health NIo. U.S (2021). Clinical Trial Results Show Novavax Vaccine Is Safe and Prevents COVID-19. NIH. https://www.nih.gov/news-events/news-releases/us-clinical-trial-results-show-novavax-vaccine-safe-prevents-covid-19.

[140] Sharma K., Koirala A., Nicolopoulos K., Chiu C., Wood N., Britton P.N. (2021). Vaccines for COVID-19: Where do we stand in 2021?. Paediatr. Respir. Rev..

[141] Diseases NIoAaI (2019). Vaccine Types. NIH. https://www.niaid.nih.gov/research/vaccine-types.

[142] ClinicalTrials.gov Study of the Safety, Reactogenicity and Immunogenicity of “EpiVacCorona” Vaccine for the Prevention of COVID-19 (EpiVacCorona) 2021. https://clinicaltrials.gov/ct2/show/NCT04527575.

[143] WHO (2021). The Sinopharm COVID-19 Vaccine: What You Need to Know. WHO. https://www.who.int/news-room/feature-stories/detail/the-sinopharm-covid-19-vaccine-what-you-need-to-know.

[144] Nicholson L.B. (2016). The immune system. Essays Biochem..

[145] Weiskopf D., Weinberger B., Grubeck-Loebenstein B. (2009). The aging of the immune system. Transpl. Int..

[146] Simon A.K., Hollander G.A., McMichael A. (2015). Evolution of the immune system in humans from infancy to old age. Proc. Biol. Sci..

[147] Chaplin D.D. (2010). Overview of the immune response. J. Allergy Clin. Immunol..

[148] Peter D., Stranding S., Livingstone C. (2006). Gray’s Anatomy. Peptide Applications in Biomedicine, Biotechnology and Bioengineering.

[149] Wiendl H., Lautwein A., Mitsdörffer M., Krause S., Erfurth S., Wienhold W., Morgalla M., Weber E., Overkleeft H.S., Lochmuller H. (2003). Antigen processing and presentation in human muscle: Cathepsin S is critical for MHC class II expression and upregulated in inflammatory myopathies. J. Neuroimmunol..

[150] Florindo H.F., Kleiner R., Vaskovich-Koubi D., Acúrcio R.C., Carreira B., Yeini E., Tiram G., Liubomirski Y., Satchi-Fainaro R. (2020). Immune-mediated approaches against COVID-19. Nat. Nanotechnol..

[151] Pušnik J., Richter E., Schulte B., Dolscheid-Pommerich R., Bode C., Putensen C., Hartmann G., Alter G., Streeck H. (2021). Memory B cells targeting SARS-CoV-2 spike protein and their dependence on CD4. Cell Rep..

[152] Prevention CfDCa (2020). Local Reactions, Systemic Reactions, Adverse Events, and Serious Adverse Events: Pfizer-BioNTech COVID-19 Vaccine. https://www.cdc.gov/vaccines/covid-19/info-by-product/pfizer/reactogenicity.html.

[153] BioNTech (2020). Update on Our COVID-19 Vaccine Development Program with BNT162b2. https://investors.biontech.de/static-files/53f0968a-279b-4f82-a2fc-d67dcb6e4e91.

[154] Wise J. (2021). Covid-19: European countries suspend use of Oxford-AstraZeneca vaccine after reports of blood clots. BMJ.

[155] Blauenfeldt R.A., Kristensen S.R., Ernstsen S.L., Kristensen C.C.H., Simonsen C.Z., Hvas A.-M. (2021). Thrombocytopenia with acute ischemic stroke and bleeding in a patient newly vaccinated with an adenoviral vector-based COVID-19 vaccine. J. Thromb. Haemost..

[156] Agency E.M. (2021). EMA’s Safety Committee Continues Investigation of COVID-19 Vaccine AstraZeneca and Thromboembolic Events. https://www.ema.europa.eu/en/news/emas-safety-committee-continues-investigation-covid-19-vaccine-astrazeneca-thromboembolic-events.

[157] Ledford H. (2021). COVID vaccines and blood clots: Five key questions. Nature.

[158] Mulligan M.J., Lyke K.E., Kitchin N., Absalon J., Gurtman A., Lockhart S., Neuzil K., Raabe V., Bailey R., Swanson K.A. (2020). Phase I/II study of COVID-19 RNA vaccine BNT162b1 in adults. Nature.

[159] Callway E. (2020). Oxford COVID Vaccine Results Puzzle Scientists. Nature.

[160] Agency MHPR (2021). Coronavirus Vaccine—Weekly Summary of Yellow Card Reporting. https://www.gov.uk/government/publications/coronavirus-covid-19-vaccine-adverse-reactions/coronavirus-vaccine-summary-of-yellow-card-reporting.

[161] Bucci E., Andreev K., Björkman A., Calogero R.A., Carafoli E., Carninci P., Castagnoli P., Cossarizza A., Mussini C., Guerin P. (2020). Safety and efficacy of the Russian COVID-19 vaccine: More information needed. Lancet.

[162] Cyranoski D. (2020). Why emergency COVID-vaccine approvals pose a dilemma for scientists. Nat. Cell Biol..

[163] Mahase E. (2020). Covid-19: What have we learnt about the new variant in the UK?. BMJ.

[164] Nagy Á., Pongor S., Győrffy B. (2021). Different mutations in SARS-CoV-2 associate with severe and mild outcome. Int. J. Antimicrob. Agents.

[165] Iyengar K.P., Jain V.K., Ish P. (2020). COVID-19 reinfection—An enigmatic public health threat. Monaldi Arch. Chest Dis..

[166] Raghav S., Ghosh A., Turuk J., Kumar S., Jha A., Madhulika S., Priyadarshini M., Biswas V.K., Shyamli P.S., Singh B. (2020). Analysis of Indian SARS-CoV-2 Genomes Reveals Prevalence of D614G Mutation in Spike Protein Predicting an Increase in Interaction With TMPRSS2 and Virus Infectivity. Front. Microbiol..

[167] Harvey W.T., Carabelli A.M., Jackson B., Gupta R.K., Thomson E.C., Harrison E.M., Ludden C., Reeve R., Rambaut A. (2021). COVID-19 Genomics UK (COG-UK) Consortium.; et al. SARS-CoV-2 variants, spike mutations and immune escape. Nat. Rev. Microbiol..

[168] Prevention CfDCa SARS-CoV-2 Variant Classifications and Definitions 2021. https://www.cdc.gov/coronavirus/2019-ncov/variants/variant-info.html.

[169] Abu-Raddad L.J., Chemaitelly H., Butt A.A. (2021). Vaccination NSGfC-. Effectiveness of the BNT162b2 Covid-19 Vaccine against the B.1.1.7 and B.1.351 Variants. N. Engl. J. Med..

[170] Cohen J. (2021). South Africa suspends use of AstraZeneca’s COVID-19 vaccine after it fails to clearly stop virus variant. Science.

[171] Callaway E. (2021). Delta coronavirus variant: Scientists brace for impact. Nat. Cell Biol..

[172] Planas D., Veyer D., Baidaliuk A., Staropoli I., Guivel-Benhassine F., Rajah M.M., Planchais C., Porrot F., Robillard N., Puech J. (2021). Reduced sensitivity of SARS-CoV-2 variant Delta to antibody neutralization. Nature.

[173] Prevention CfDCa (2021). Delta Variant: What We Know About the Science. CDC. https://www.cdc.gov/coronavirus/2019-ncov/variants/delta-variant.html.

[174] Bernal J.L., Andrews N., Gower C., Phil D., Gallagher E., Simmons R., Thelwall S., Stowe J., Tessier E., Groves N. (2021). Effectiveness of Covid-19 Vaccines against the B.1.617.2 (Delta) Variant. N. Engl. J. Med..

[175] Sanderson K. (2021). COVID vaccines protect against Delta, but their effectiveness wanes. Nat. Cell Biol..

[176] Packham H., Nina G.D., Charles R., Radziszewska A., Ciurtin C., Wedderburn R.L., Rosser E.C., Deakin C., Webb K. (2020). Sex-bias in COVID-19: A meta-analysis and review of sex differences in disease and immunity. Res. Sq..

[177] Jin J.M., Bai P., He W., Wu F., Liu X.F., Han D.M., Liu S., Yang J.-K. (2020). Gender Differences in Patients With COVID-19: Focus on Severity and Mortality. Front. Public Health.

[178] Scully E.P., Haverfield J., Ursin R.L., Tannenbaum C., Klein S.L. (2020). Considering how biological sex impacts immune responses and COVID-19 outcomes. Nat. Rev. Immunol..

[179] Bouman A., Heineman M.J., Faas M.M. (2005). Sex hormones and the immune response in humans. Hum. Reprod. Update.

[180] Ecker J.W., Kirchenbaum G.A., Pierce S.R., Skarlupka A.L., Abreu R.B., Cooper R.E., Taylor-Mulneix D., Ross T.M., Sautto G.A. (2020). High-Yield Expression and Purification of Recombinant Influenza Virus Proteins from Stably-Transfected Mammalian Cell Lines. Vaccines.

[181] Reshma N.J., Istvan T., Maruisz S. (2018). 12-Peptide-Based Vaccines. Peptide Applications in Biomedicine, Biotechnology and Bioengineering.

